# The Bangladesh Risk of Acute Vascular Events (BRAVE) Study: objectives and design

**DOI:** 10.1007/s10654-015-0037-2

**Published:** 2015-05-01

**Authors:** Rajiv Chowdhury, Dewan S. Alam, Ismail Ibrahim Fakir, Sheikh Daud Adnan, Aliya Naheed, Ishrat Tasmin, Md Mostafa Monower, Farzana Hossain, Fatema Mahjabin Hossain, Md Mostafizur Rahman, Sadia Afrin, Anjan Kumar Roy, Minara Akter, Sima Akter Sume, Ajoy Kumer Biswas, Lisa Pennells, Praveen Surendran, Robin D. Young, Sarah A. Spackman, Khaled Hasan, Eric Harshfield, Nasir Sheikh, Richard Houghton, Danish Saleheen, Joanna MM Howson, Adam S. Butterworth, Cardiology Research Group, Rubhana Raqib, Abdulla Al Shafi Majumder, John Danesh, Emanuele Di Angelantonio

**Affiliations:** Cardiovascular Epidemiology Unit, Department of Public Health and Primary Care, University of Cambridge, Cambridge, UK; Chronic Non-communicable Disease Unit, International Centre for Diarrhoeal Disease Research, Dhaka, Bangladesh; National Institute of Cardiovascular Disease, Dhaka, Bangladesh; Department of Biostatistics and Epidemiology, Perelman School of Medicine, University of Pennsylvania, Philadelphia, PA USA; Wellcome Trust Sanger Institute, Hinxton, Cambridge, UK

**Keywords:** Non-communicable diseases, Cardiovascular disease, Coronary heart disease, Myocardial infarction, Risk factors, Arsenic, Genetics, Bangladesh, South Asia, BRAVE

## Abstract

During recent decades, Bangladesh has experienced a rapid epidemiological transition from communicable to non-communicable diseases. Coronary heart disease (CHD), with myocardial infarction (MI) as its main manifestation, is a major cause of death in the country. However, there is limited reliable evidence about its determinants in this population. The Bangladesh Risk of Acute Vascular Events (BRAVE) study is an epidemiological bioresource established to examine environmental, genetic, lifestyle and biochemical determinants of CHD among the Bangladeshi population. By early 2015, the ongoing BRAVE study had recruited over 5000 confirmed first-ever MI cases, and over 5000 controls “frequency-matched” by age and sex. For each participant, information has been recorded on demographic factors, lifestyle, socioeconomic, clinical, and anthropometric characteristics. A 12-lead electrocardiogram has been recorded. Biological samples have been collected and stored, including extracted DNA, plasma, serum and whole blood. Additionally, for the 3000 cases and 3000 controls initially recruited, genotyping has been done using the CardioMetabochip+ and the Exome+ arrays. The mean age (standard deviation) of MI cases is 53 (10) years, with 88 % of cases being male and 46 % aged 50 years or younger. The median interval between reported onset of symptoms and hospital admission is 5 h. Initial analyses indicate that Bangladeshis are genetically distinct from major non-South Asian ethnicities, as well as distinct from other South Asian ethnicities. The BRAVE study is well-placed to serve as a powerful resource to investigate current and future hypotheses relating to environmental, biochemical and genetic causes of CHD in an important but under-studied South Asian population.

**Electronic supplementary material:**

The online version of this article (doi:10.1007/s10654-015-0037-2) contains supplementary material, which is available to authorized users.

## Introduction

Coronary heart disease (CHD), of which myocardial infarction (MI) is an important manifestation, remains the single leading cause of death worldwide [1]. The majority of premature CHD deaths now occur in low- and middle-income countries [1–3]. In particular, South Asia has recorded the highest number of life-years lost due to premature CHD, a situation which reflects both the region’s large population and the relatively young age at which CHD death occurs in this population [4]. Furthermore, while age-standardised CHD mortality rates have decreased during recent decades in many high-income countries, they have continued to increase in South Asia [5]. Nevertheless, there is limited evidence available about the determinants of CHD in South Asia, even though it could contribute importantly to scientific understanding and to the development of appropriate strategies for the prevention and control of CHD [6].

Bangladesh has experienced steep and sustained increases in the incidence of CHD and other cardiovascular conditions during recent decades [7]. Bangladesh is a country with a population of over 160 million [8], yet it is one of least studied major countries with regard to cardiovascular disease [9]. The burden of CHD in Bangladeshis is not just of local public health concern. For example, CHD mortality has been reported to be more than two times higher among Bangladeshis living in western regions compared to native western populations [10, 11]. The burden of CHD in Bangladeshis living in western regions is also higher than that of most other migrant groups, including South Asians from India and Pakistan [9]. An important challenge is, therefore, to establish informative epidemiological resources in a rigorous yet cost-effective manner to evaluate risk factors among Bangladeshis.

The present report provides a description of objectives and methods used in the establishment of the Bangladesh Risk of Acute Vascular Events (BRAVE) study. It also describes the baseline characteristics of the study population recruited so far, and outlines the rationale for the study’s further development.

## Methods

**Fig. 1:**
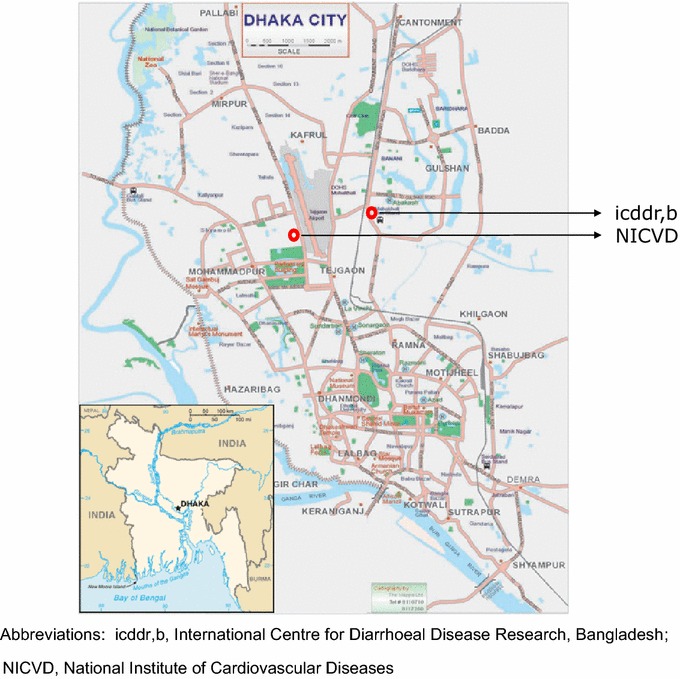
Location of the collaborating and recruitment centres in Dhaka

The BRAVE study was established in 2011 by the Department of Public Health and Primary Care at the University of Cambridge (the study’s international coordinating centre), in collaboration with the Chronic Non-communicable Disease Unit at the International Centre for Diarrhoeal Disease Research, Bangladesh (icddr,b) and the National Institute of Cardiovascular Disease (NICVD) in Bangladesh. The icddr,b is the project’s national collaborating centre and houses the local laboratory facilities for the study (Fig. 1). The NICVD, Bangladesh’s largest cardiology care centre, treats MI patients from Dhaka (the capital city; population ~15 million) as well as from surrounding semiurban and rural areas.

BRAVE has received approval from the relevant research ethics committee of each of the institutions involved in participant recruitment. Written informed consent has been obtained from each participant prior to recruitment, including for use of stored samples for biochemical, genetic and other analyses. Data collected in this research are subject to the core data protection principles and requirements of the UK Data Protection Act 1998. The investigators and institutional review boards are committed to ensure that research is conducted according to the latest version of the Declaration of Helsinki, the Universal Declaration on the Human Genome and Human Rights adopted by UNESCO, and other relevant legislation.

### Study design and participants

**Fig. 2:**
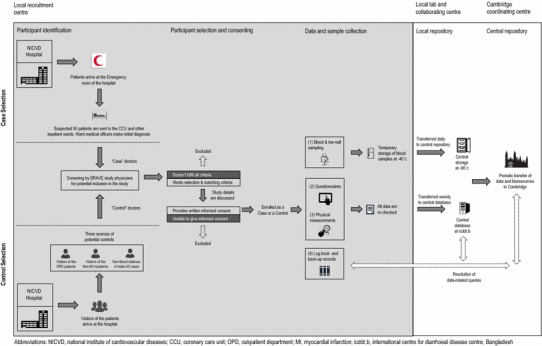
BRAVE study flow diagram of surveillance, enrolment and data collection

BRAVE is a retrospective case–control study of acute MI (Fig. 2). Following screening by medically-qualified research officers, patients (male or female; aged at least 20 years) admitted to the emergency rooms of the NICVD hospital are eligible for inclusion as MI cases if they fulfil all of the following criteria: (1) presentation at the hospital within 48 h of the onset of sustained clinical symptoms suggestive of MI lasting longer than 20 min; (2) presence of ECG changes indicative of MI (i.e., new pathologic Q waves, at least 1 mm ST elevation in any 2 or more contiguous limb leads or a new left bundle branch block, or new persistent ST-T wave changes diagnostic of a non-Q wave MI); (3) increased cardiac troponin-I (cTnI) levels [12]; (4) no previous cardiovascular disease, defined as self-reported history of angina, MI, coronary revascularisation, transient ischaemic attack, stroke, other cardiovascular disease or evidence of CHD on prior ECG, or in other medical records (eTable 1); and (5) not concurrently hospitalised for any other cardiovascular disease events.

Controls were individuals without a previous self-reported history of cardiovascular disease (as defined above) drawn from individuals concurrently identified in the same hospital as index cases, and recruited in the following order of priority: (1) visitors of patients attending the out-patient department; (2) visitors of in-patients who are not part of the BRAVE study; and (3) visitors of index MI cases who are not their blood relatives. Controls have been recruited within 48 h of recruiting index cases, and they are “frequency-matched” to cases by sex and age (in 5-year age bands). Participants have not been enrolled if any of the following features are evident: (1) a history of acute viral or bacterial infection in the previous 2 weeks; (2) documented chronic conditions, such as malignancy, any chronic infection, leprosy, malaria, tuberculosis or other bacterial/parasitic infections, chronic inflammatory disorders, chronic hepatitis or chronic kidney disease on past medical history; (3) a recent history of any surgery; (4) pregnancy; or (5) unable to provide consent.

### Questionnaire administration and physical measurements

Research medical officers in BRAVE have administered pre-piloted epidemiological questionnaires to participants that sought over 350 items of information in relation to demographic characteristics, lifestyle factors (e.g., tobacco and alcohol consumption, dietary intake and physical activity), personal and family medical history, and medication usage (Table 1; a copy of the study questionnaire is available at www.phpc.cam.ac.uk/ceu/research/brave). To collect information on smoking patterns distinctive to South Asian populations, BRAVE has adapted a population-specific questionnaire used in other studies of South Asians [13]. Additionally, a food-frequency questionnaire previously developed and validated in Bangladesh [14] has been adapted and further modified for BRAVE (Table 2). This questionnaire estimates standard portion size assigned to each food item. Using standardized procedures and equipment; trained research nurses have obtained measurements of height, weight, waist and hip circumference, systolic and diastolic blood pressure, and heart rate. Waist circumference has been assessed over the abdomen at the widest diameter between the costal margin and the iliac crest, and hip circumference has been assessed at the level of the greater trochanters (i.e., the widest diameter around the buttocks). For both cases and controls, anthropometric measurements have been performed in a standing position. Study medical officers have recorded a standard 12-lead ECG.

**Table 1:** Summary of questionnaire-based information collected

Characteristics	Availability of information
Demographic and lifestyle factors	Age at onset, gender, tobacco use (cigarette or non-cigarette), exposure to environmental tobacco, detailed dietary habits, and levels of physical activity
Socio economic factors	Education, occupation, objects owned, total income, marital status, family size, indoor air pollution, sources of drinking water
Economic burden of MI	Personal cost associated with the current event (treatment and accommodation), source of referral, transportation costs, costs of attendants, sources of payment
Knowledge and awareness	Levels of knowledge and perception of cardiovascular disease, risk factors (such as smoking, diabetes and blood pressure), prevention and practices would be assessed among controls
Psychosocial factors	Stress at work and home, social support, depression and life events; sleeping habits
Women’s health	Use of hormonal contraceptives, menstrual and pregnancy history
Medical history	Cardiovascular disease, hypertension, diabetes mellitus, atrial fibrillation, cancer, hypercholesterolemia, current medication use including regularity of antihypertensive intake, other vascular disease, infectious disease, major surgery and family history
Physical measurements	Blood pressure at baseline, heart rate, height, weight, waist and hip circumferences
Coronary assessment	Time to initiate thrombolysis, detailed recording of cardiac work-up (e.g. ECG changes/ECHO), reversal of symptoms and ST segment elevation by >50 % on streptokinase infusion, cardiac marker (Troponin I)
MI Subtype	Based on clinical assessment and ECG/ECHO findings subtypes of MI are recorded (such as, anterior, antero-septal, inferior, lateral, posterior, right ventricle or non ST elevated MI)
Course in hospital and status	Treatments given, adverse events (if any), outcomes in hospital

*ECG* electrocardiogram, *ECHO* echocardiogram, *MI* myocardial infarction

**Table 2:** Information collected on dietary intake

Food group	Routinely recorded information	Relevant key information recorded to reflect local habits
Rice	Type and amount of rice	Steamed rice (hand pounded) e.g., sada bhat; mixed rice such as biriyani, polau and tehari
Bread	Type and numbers of bread slices	Wheat bread e.g., ruti; recording type flour bread, e.g., chapatti; oil coded, e.g., porotha or luchi; white bread such as pauruti
Other carbohydrates	Potato and sugar	Consumption of aloo and chini
Meat and poultry	Chicken, eggs, liver, beef, mutton, and lamb	Type of chicken, e.g., caged (farmed) or free-range (deshi); method of cooking, e.g., grilled or curried
Fish	Type and amount of fish	Source such as sweet or saline water fish
Dairy	Milk, butter and margarine and other dairy products	Misti (dairy-based dessert); doi (yogurt-based dessert)
Vegetables	Types and amount	Cooked or raw vegatables; green leafy, yellow, cruciferus, salad vegetable subtypes
Pulses	Lentil subtypes	Local lentil produce e.g., muger dal, musurir dal, buter dal
Spices	Types and amount used in traditional cooking	Cumin (jeera), Ginger (ada), turmeric (holud) and garlic (roshun)
Fruits	Types and amount	Seperately for locally grown and imported fruits
Fast foods	Types and amount	Source (bought or home-made); local recipe (e.g., puri, singara, samosa, kabab) and Western recipe (e.g., burger, pizza, sandwich, cakes)
Drinks	Tea, coffee, soft drinks, alcohol	Local beverages such as lassi; local alcoholic preparations; type of sugar-sweetened beverages, such as carbonated and noncarbonated drinks
Cooking medium	Cooking oil (recording type)l	Common local vegetable oil such as palm oil; refined vegetable oil such as Banaspati or dalda; purified butter oil (such as ghee); oil purchase circumstance such as bought from open (unpurified) sources (“khola tel”) versus as closed container

### Data entering, transfer and checking

Two staff involved with data entry, working independently of each other, have entered into a central database information extracted from the questionnaire, physical measurements and ECG recording. Copies of this database have been held securely, both at icddr,b and at the Department of Public Health and Primary Care, University of Cambridge. Access to study data is available only to the principal investigators and designated data managers working under their direct supervision. Data have been transferred monthly to the coordinating centre in Cambridge, UK, where they have been checked for internal consistency; queries have been referred back for clarification to the team based at icddr,b. Computer-generated detailed summary tabulations have been reviewed weekly by a designated study data manager in Cambridge to help monitor the study’s progress. Paper copies of completed questionnaires have been securely stored at the icddr,b. From mid-2015, data will be collected in a “paper-less” approach that involves electronic data collection using a bespoke android interface operated through hand-held tablet devices.

### Collection of biological samples

Non-fasting blood samples (with the time since last meal recorded) have been drawn by trained study staff nurses from each participant and centrifuged (@10,000 rpm for 10 min) within 45 min of venepuncture. For MI cases, blood sampling is conducted within 48 h of the onset of clinical symptoms (time since onset of pain is recorded) and prior to the administration of any thrombolytic medication. As the blood sample is typically obtained from MI cases while they are in a recumbent position (e.g., at about 45°), the sampling in the controls has also been carried out in the same manner to limit the possibility of systematic differences. A total of 24 ml of whole blood has been drawn from each participant in 2 × 6 ml serum tubes and 2 × 6 ml EDTA tubes (eFigure 1). Isolated serum, EDTA plasma and whole blood samples have been transported daily to the local laboratory where they are stored in cryogenic vials at −80 °C, following temporary storage for few hours at the laboratory of the recruitment hospital at −40 °C. From January 2013, toenail clippings have been taken from all ten toes of each participant and initially stored in labelled zip-lock bags at room temperature. Biological samples have been stored in long-term repositories in both Cambridge, UK, and in Dhaka, Bangladesh.

### DNA quality and quantification

DNA has been extracted by the Laboratory of the Government Chemist (LGC) Genomics (Herts, UK) from whole blood samples using a validated silica-based method (eFigure 2) [15]. In the initial 6000 extractions (average yield 100–120 µg per sample), 99.2 % of samples have had concentrations high enough for genotyping (≥50 ng/μl) and all samples showed 260/280 ratios in the expected range, indicating that the purity of the extracted DNA is high. To minimise systematic biases, stock plates were generated and have been used to generate plates of DNA for genotyping, which contain a mixture of cases and controls along with the negative and positive controls (an empty well and a sample with known genotype) designed to address genotyping quality control, plate identification and orientation. Samples have been genotyped for three single nucleotide polymorphisms (SNPs) to check DNA quality and participant sex across all the samples. 100 % genotyping success rates have been observed and there have been no gender mismatches, showing that the DNA extraction, storage, labelling and transport processes already in place are capable of producing high quality genetic data.

### Initial measurements

For the initial approximately 4000 MI cases and the initial approximately 4000 controls, total cholesterol and triglycerides have been measured using an enzymatic colorimetric test (Roche Diagnostic), high-density lipoprotein cholesterol (HDL-C) has been measured using a homogenous enzymatic colorimetric assay (Roche Diagnostic HDL-C plus 3rd generation), and low-density lipoprotein cholesterol (LDL-C) has been measured using a homogenous enzymatic colorimetric assay (Roche Diagnostic LDL-C plus 2nd generation). All lipid assays have been done with Roche automated clinical chemistry analyzer (Hitachi 902, Hitachi Ltd, Tokyo, Japan). For the initial approximately 3000 MI cases and initial approximately 3000 controls, genotyping has been done using the “CardioMetabochip+ array” (containing ~210,000 SNPs) and the “Exome+ array” (containing ~420,000 single nucleotide variants [SNVs], mostly low frequency and rare coding variants), both of which are manufactured by Illumina (San Diego, CA, USA). The content of these arrays has been described previously [16, 17]. Pilot studies have commenced to assess arsenic and other toxic metal (such as lead, cadmium, and copper) in toenail and whole blood samples using inductively coupled plasma mass spectrometry [18].

### Plans for expansion

By early 2015, the ongoing BRAVE study had recruited over 5000 confirmed first-ever MI cases and 5000 controls. The main objectives of the study’s next stages are: to expand the study to 10,000 acute MI cases and 10,000 controls; to enrich the bioresource in various ways in order to increase its scientific value; and to accelerate harvesting of its biological resources (Table 3). Serial blood measurements have been done within the first 24 h of hospital admission from an initial 100 acute MI cases, enabling quantification and correction for potential distortions due to onset acute MI. Furthermore, serial epidemiological questionnaires, anthropometry, and blood sampling is planned in about 500 participants approximately 2–3 years following the initial visit, enabling quantification of and correction for regression dilution [19]. Such re-surveys should help further validate various questionnaires developed in BRAVE.

**Table 3:** Biological measurements planned or in progress

Approach	Analytical strategy
(1) *Genomics*	
CardioMetabochip + array	~210,000 SNPs of interest for cardiovascular disease traits.
Exome + array	~420,000 SNVs, mostly low frequency and rare coding variants
Sequencing	High-depth sequencing
(2) *Biomarkers*	
Cardiometabolic analytes	Total cholesterol, HDL-C, LDL-C, triglycerides, HbA1c, Lp(a)
Toxic heavy metals	Total arsenic, arsenic metabolites, copper, lead, cadmium, and mercury

*SNPs* single nucleotide polymorphisms, *SNVs* single nucleotide variants, *HDL-C* high-density lipoprotein cholesterol, *LDL-C* low-density lipoprotein cholesterol, *HbA1c* haemoglobin A1c, *Lp* (*a*) lipoprotein (a)

### Statistical analysis

This report includes analyses for the initial approximately 4500 acute MI cases and 4500 controls, for whom lipid measurements are available in 4188 cases and 4130 controls. For baseline variables, mean [standard deviation (SD)] values and frequencies were calculated separately for MI cases and controls and compared using *t* test for continuous variables or *Chi*-squared test for categorical variables. Genetic ancestry of the BRAVE participants was evaluated with principal component analysis, using 19,931 SNPs contained on the Exome+ array. SNPs were selected to have a minor allele frequency >0.05 in South Asians, not to be in strong linkage disequilibrium (R^2^ < 0.20) with one another, and not to relate to known CHD-associated regions [20]. To examine the ancestry of the BRAVE samples in a global context, 5756 participants in BRAVE and 2504 participants from 26 other populations in the 1000 Genomes project [21] were included in a principal component analysis. A South Asian specific principal component analysis of BRAVE and the five South Asian populations from the 1000 Genomes (one of which comprises Bengalis from Bangladesh) was also performed.

Analyses were performed using either Stata (version 13, StataCorp, College Station, TX) or R (http://www.R-project.org/). Statistical approach for future analyses will be developed following relevant guideline statements about reporting standards for observational studies (e.g., STROBE [22], NCI/NHGR working group [23]).

## Results

For the initial 4500 MI cases and 4500 controls, the mean delay from blood sample collection (with immediate chilling) to sample separation (with immediate freezing) was about 15 min. The median (interquartile range) time recorded between the time of participant’s last meal and blood collection was 4.2 (2.4–7.3) h. 48 % of participants reported living in urban areas, and 52 % reported living in rural areas. 93 % of participants reported being married. About 35 % of the participants reported having at least 10 years of formal education. The proportion with such levels of formal education was higher in men than women (38 vs. 12 %, *p* < 0.001), and in people from urban rather than rural areas (44 vs. 27 %, *p* < 0.001).

The mean age (SD) of MI cases was 53 (10) years. Almost half (46 %) of the cases were aged 50 or younger. 88 % of the cases were male. The median interval recorded between the reported onset of MI symptoms and hospital admission was 5 h. As would be expected, the following risk factors were more prevalent in cases than controls: tobacco consumption, history of diabetes and of hypertension, and parental family history of MI. Total and LDL cholesterol levels were higher in cases than controls, whereas HDL cholesterol levels were lower in cases than controls (Table 4).

**Table 4:** Baseline characteristics of the initial participants recruited

Characteristic	Cases		Controls		*p* value^*†*^
	N	Mean (SD) or %	N	Mean (SD) or %	
Age (years)	4500	52.6 (10.4)	4500	50.4 (10.1)	Matched
*Sex* (%)					
Males	3950	88	3935	87	
Females	550	12	565	13	Matched
*Tobacco consumption (*%)					
Never	630	15	1383	31	
Ex	246	6	301	7	
Current	3224	79	2811	63	<0.001
*History of hypertension* (%)					
Yes	1090	24	470	10	
No	3404	76	4027	90	<0.001
*History of diabetes* (%)					
Yes	778	17	359	8	
No	3716	83	4138	92	<0.001
*Family history of MI* ^‡^ (%)					
Yes	597	13	267	6	
No/Unknown	3903	87	4233	94	<0.001
*Location* (%)					
Urban	1891	47	2183	49	
Rural	2142	53	2282	51	0.065
*Level of education reached* (%)					
No schooling	1390	34	1576	35	
Primary	1269	31	1297	29	
Secondary	944	23	1107	25	
Vocation/University	454	11	518	12	0.093
Blood lipids measurements					
Total cholesterol (mmol/l)	4188	5.14 (1.14)	4130	4.77 (1.00)	<0.001
LDL-C (mmol/l)	4188	3.19 (1.03)	4128	2.76 (0.86)	<0.001
HDL-C (mmol/l)	4188	0.85 (0.22)	4130	0.87 (0.22)	<0.001

^†^ *p* *value* obtained from *t* test for continuous variables or Chi squared test for categorical variables. ^‡^ Mother and/or father

*HDL-C* high-density lipoprotein cholesterol, *LDL-C* low-density lipoprotein cholesterol, *MI* myocardial infarction

**Fig. 3:**
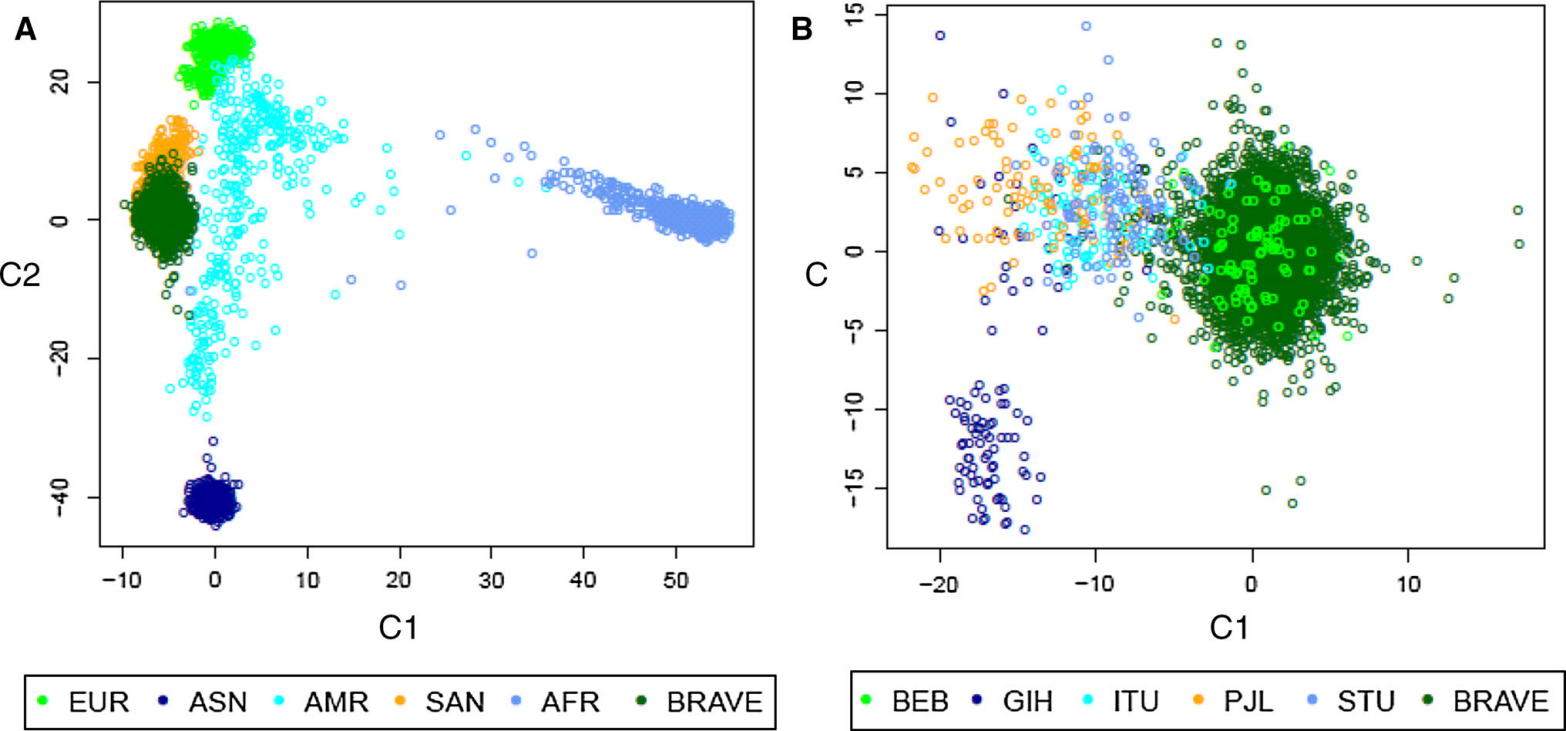
Genetic ancestry in the BRAVE population derived from Principal Component Analysis. The figures above indicate that Bangladeshis (i.e. the BRAVE study participants and those in the BEB 1000 Genomes study) cluster separately from major non-South Asian populations (**a**) and other South Asian (**b**) ethnic groups. BRAVE indicates the Bangladeshis from BRAVE study participants. The colours of points refer to the self-reported ethnicities in the BRAVE (n = 5756 and the 1000 Genomes (n = 2504) study participants: SAN, South Asians; ASN, East Asians; EUR, Europeans; AMR, admixed Americans; AFR, Africans; BEB, Bengalis in Bangladesh (Non-BRAVE); GIH, Gujrati Indians in Houston, US; ITU, Indian Telegus in the UK; PJL, Pakistani Punjabis in Pakistan; STU, Sri Lankan Tamils in the UK. Scatterplot are of the first 2 principal components. C1, first principal component; and C2, second principal component

Comparison with the 1000 Genomes panel of populations showed that the Bangladeshi population is genetically distinct from major non-South Asian ethnic groups. This is suggested by the separate clustering on the scatterplot of principal components (Fig. 3a). Furthermore, Bangladeshis clustered distinctly from several other South Asian ethnicities in the 1000 Genomes panel (Fig. 3b), and were perhaps genetically closest to (though still distinct from) Sri Lankan Tamils.

## Discussion

We have established the first large epidemiological bioresource for the study of MI and related traits in Bangladesh, demonstrating the feasibility and validity of the study methods we have used. The initial findings have highlighted the striking early-onset nature of MI in Bangladesh, with nearly half of the cases recruited into BRAVE aged 50 years or younger. The enduring value of large case–control studies of CHD to investigate genetic and certain environmental factors has been demonstrated by previous efforts, such as by the International Studies of Infarct Survival (ISIS) collaboration (14,000 acute MI cases and 32,000 controls in the UK) [24] and by the INTERHEART study (15,000 acute MI cases and 15,000 controls from 52 countries) [25].

Until BRAVE, however, the largest study of CHD in Bangladesh comprised only 228 MI cases and only 238 controls as a component of the INTERHEART study [25]. Furthermore, whereas INTERHEART assessed cardiovascular risk factors common to many countries, the complementary approach in BRAVE has been to focus on these common risk factors plus a more detailed assessment of risk factors distinctive to Bangladesh. This focus should help us to address various unmet strategic needs, such as those described below.

The first need is to estimate the impact of modifiable lifestyle factors, since such findings could be of considerable relevance to the prevention and control of cardiovascular disease in Bangladesh. For example, little is known about the relevance to CVD of distinctive and widespread practices in Bangladesh and other South Asian countries, such as use of smokeless tobacco (such as *jarda* or *gul*) and different types of oil used in cooking (such as palm oil or *banaspati*).

Second, there is a need to determine the relevance to CHD of arsenic contamination in drinking water in Bangladesh, described by the World Health Organization (WHO) as “the largest mass poisoning of a human population in history” [26]. For, in addition to the established carcinogenic effects of chronic arsenic exposure, evidence is emerging of an association between arsenic exposure and common manifestations of cardiovascular disease, such as MI [27]. However, studies of arsenic contamination and CHD in Bangladesh have hitherto been limited, so far comprising a single report involving only about 100 CHD outcomes ascertained through verbal autopsy [28]. Consequently, it has not been possible to establish reliably whether or not arsenic exposure is a causative risk factor for CHD, nor has it been possible to characterise the shape of any dose–response relationship. For this reason, the WHO and US Environmental Protection Agency have stated that cardiovascular disease cannot be considered a relevant consideration in defining maximum arsenic contaminant levels [29]. As a related issue, there is a need to assess potential joint effects of arsenic and other metals (such as lead, cadmium, mercury and copper) on CHD risk. These metals commonly co-occur with arsenic in groundwater in the deltaic environments of Bangladesh [30]. Hence, even more powerful studies are required to study such joint effects reliably than to assess the relevance to CHD of arsenic alone. We plan, therefore, to continue expansion of recruitment in BRAVE for some years and to continue collection of relevant biological samples (such as toenails and plasma) in order to facilitate this assessment.

A third need is to create a resource of specific relevance to people of Bangladeshi ancestry in order to advance understanding of the genetic determination of CHD and a variety of related complex traits in this population. In recent years, a limited number of major genetic CHD bioresources involving (non-Bangladeshi) South Asians have emerged. Notable examples include the Pakistan Risk of Myocardial Infarction Study (PROMIS, a study of over 15,000 MI cases and over 15,000 controls in urban Pakistan [13], led by some of the investigators in BRAVE), and the London Life Sciences Prospective Population Study (LOLIPOP, a study of over 3500 CHD cases and over 4000 controls [31], predominantly of Indian and Pakistani descent, living in London, UK). BRAVE complements these studies by providing a resource of comparable size and scope in Bangladeshis. Indeed, the results of the principal component analysis in BRAVE support the suggestion that Bangladeshis are genetically distinct from the other South Asian ethnicities. Furthermore, BRAVE complements studies of Bangladeshis resident in Western countries (such as the recently-launched East London Genes & Health Study [32], which proposes to involve people of Bangladeshi- and Pakistani-origin who live in London and which aims to recruit participants irrespective of CHD status).

Genetic studies of people of South Asian ancestry should help to identify both population-specific as well as genetic risk factors shared by people from different continental ancestries (“cosmopolitan” risk factors). Furthermore, there is evidence to suggest the potential scope for important ethnic-specific genetic discoveries for cardiovascular disease in South Asians. For example, a 25 bp deletion in *MYBPC3* associated with hypertrophic cardiomyopathy and a sevenfold increase in the risk of heart failure was discovered in South Asians, whereas it would have probably remained undetected if studies were confined to other ancestries (since this variant was nearly absent in the other 20 non-South Asian populations studied [33]). Additionally, a variant in the *SGCG* gene has been associated with type 2 diabetes in Punjabi Sikhs from northern India, but not with type 2 diabetes in non-South Asians or with type 2 diabetes in other ethnic groups from South Asia [34]. The discovery of ethnic-specific genetic risk factors for Bangladeshis (and, more generally, other South Asians) should be facilitated by the use of next generation sequencing technologies. By contrast, the ability of most previous studies of South Asians to find population-specific genetic risk factors has been constrained by the use of gene arrays based on catalogues of genetic variation mostly discovered in Europeans, East Asians, and African Americans. Nevertheless, the use of such arrays has enabled PROMIS and LOLIPOP to contribute importantly to the discovery of over 25 cosmopolitan loci for CHD [17, 35, 36] and type 2 diabetes [34, 37, 38], showing that there are genetic risk factors for cardiometabolic conditions that apply to people of South Asian ancestry and to people of European ancestry.

The strengths and potential limitations of the BRAVE study merit consideration. Retrospective case–control studies of MI can usefully complement prospective studies because the former involve ascertainment of exposure information and blood sampling of people who have already developed MI and a comparable group of controls without MI, which enables rapid and cost-effective accrual of large numbers of relevant cases, especially in low and middle-income countries. Furthermore, retrospective studies are often able to include large numbers of individuals who have developed the disease at younger ages, when associations with risk factors are often stronger. Hence, case–control studies can provide particularly sensitive tests of certain hypotheses. As demonstrated by the Wellcome Trust Case–Control Consortium and many subsequent case–control studies, such studies can powerfully and efficiently facilitate genetic discovery and can quantify and robustly correct for any population structure [34–40].

Nevertheless, particularly in relation to non-genetic hypotheses, retrospective case–control studies may be liable to potential biases, such as selection bias, recall bias, and reverse causality. Hence, following the example of previous case–control studies of acute MI [13, 24, 25], we have involved various measures in the current study to help reduce such potential biases. For example, to reduce the scope for selection bias, we have sampled controls from approximately the same source population as the cases. To reduce the scope for recall bias, we have conducted an incident case–control study of acute MI and sought information from cases within hours of the index event. To reduce the scope for reverse causality, we intend to focus efforts on biomarkers not liable to change immediately after acute MI, such as glycosylated haemoglobin and lipoprotein (a). We acknowledge that our study may have limited power to study female-specific associations of risk factors with MI, as only about 12 % of the MI cases recruited so far have been women.

In conclusion, BRAVE is a large epidemiological bioresource to investigate the determinants of CHD and of related traits in Bangladesh. It should help to hasten discovery of disease-causing pathways and to inform appropriate disease prevention strategies in Bangladesh and beyond.

## Electronic supplementary material

Supplementary material 1 (DOCX 88 kb)
