# Supplementary material for: The Bangladesh Risk of Acute Vascular Events (BRAVE) Study: objectives and design
Source: Eur J Epidemiol. 2015 May 1;30(7):577–87. doi: 10.1007/s10654-015-0037-2 (PMC4516898; doi:10.1007/s10654-015-0037-2)
Supplement: Supplementary file 1 — Supplementary material 1 (DOCX 88 kb) [file 10654_2015_37_MOESM1_ESM.docx]

**Supplementary Tables and Figures**

**eTable 1.** Self-Reported cardiovascular conditions and ICD-10 Codes

|  |  |
| --- | --- |
| Cardiovascular condition | ICD-10 code |
|  |  |
| Angina | I20 |
| Myocardial infarction | I21-I25 |
| Transient ischemic attack | G45 |
| Stroke | I60-I69 |
| Other cardiovascular diseases | I30-52 |
|  |  |

Blood centrifuged within 45 minutes and is split into

four aliquots in the recruitment centre

1.5 ml

**icddr,b**

Two EDTA tubes are stored at

4

?

c at the recruitment centre

0.5 ml of serum is

Used for the analyses

of established lipid markers

6ml

6ml

6ml

6ml

**Serum Tubes**

**EDTA Tubes**

Whole blood

Serum

1.5 ml

1.5 ml

1.5 ml

Stored at

-

70 C at the icddr,b

and shipped on dry ice to Cambridge on

monthly basis for subsequent analyses

Transported daily to icddr,b

laboratory in a temperature

-

monitored ice box

**NICVD**

**NICVD**

Stored at

-

70 C at the icddr,b

And shipped on dry ice to Cambridge on

monthly basis for subsequent analyses

**eFigure 1.**

Summary of procedures for processing, transportation and

storage of BRAVE biological samples
